# Enhanced Biomethane Conversion and Microbial Community Shift Using Anaerobic/Mesophilic Co-Digestion of Dragon Fruit Peel and Chicken Manure

**DOI:** 10.3390/biology15010083

**Published:** 2025-12-31

**Authors:** Xiaojun Zheng, Suyun Liu, Shah Faisal, Adnan Khan, Muhammad Ihsan Danish, Abdul Rehman, Daolin Du

**Affiliations:** 1 School of the Environment and Safety Engineering, Key Laboratory of Zhenjiang, Jiangsu University, Zhenjiang 212013, China; xjzheng@ujs.edu.cn (X.Z.); 19962186425@163.com (S.L.); 2 Jiangsu Collaborative Innovation Center of Technology and Material of Water Treatment, Suzhou University of Science and Technology, Suzhou 215009, China; 3 Department of Environmental Engineering, School of Architecture and Civil Engineering, Chengdu University, Chengdu 610106, China; shahfaisal@cdu.edu.cn; 4 Co-Innovation Center for Sustainable Forestry in Southern China, College of Life Sciences, Nanjing Forestry University, Nanjing 210037, China; adnanchina@njfu.edu.cn; 5 Department of Environmental Science, Shaheed Benazir Bhutto University, Sheringal 18000, Khyber Pakhtunkhwa, Pakistan; m.ihsandanish@sbbu.edu.pk; 6 Jingjiang College, Institute of Environment and Ecology, School of the Environment and Safety Engineering, Key Laboratory of Zhenjiang, School of Emergency Management, School of Agricultural Engineering, Jiangsu University, Zhenjiang 212013, China

**Keywords:** anaerobic digestion, biogas, renewable energy, waste valorization, process stability

## Abstract

Biogas and methane generated from the anaerobic digestion (AD) of organic waste present a highly effective alternative to fossil fuels. The study assessed using dragon fruit peel (DFP) as a co-substrate to enhance chicken manure (CM) biodegradability and stabilize the AD process for methane during co-digestion. The biochemical methane potential assays were conducted at mono-controls (CM and DFP) and co-digestion at CM-75:DFP-25, CM-50:DFP-50, and CM-25:DFP-75. Compared to the controls, mono-digestion produced 103.3 mL/g of volatile solids (VSs) of CM and 34.6 mL/g VS of DFP, while all treatment groups of co-digestion exhibited an increase in methane production. The highest yield was 180.3 mL/g VS at CM-25:DFP-75 (74.6% and 421.1% increase relative to mono-digestions of CM and DFP, respectively), followed by 148.3 mL/g VS at CM-50:DFP-50 (43.6% higher than CM) and 116.7 mL/g VS at CM-75:DFP-25 (13% higher than CM). Process stability at the optimal DFP co-substrate ratio (CM-25:DFP-75) was confirmed by total volatile fatty acid (VFA) conversion, as below 0.5 g/L VFAs were observed at the end of incubation, indicating highly acceptable performance. The relative abundance of *Bacteroidetes* and *Bacillota* in the treatment groups was higher as compared to the control reactors, correlating with enhanced substrate hydrolysis and VFA production. Moreover, the enrichment of acetoclastic methanogens Methanosarcina and Methanosaeta in co-digesters at CM-25:DFP-75 was associated with the efficient degradation of acetic acid and propionic acid, which aligns with the observed increase in methane yield. The study enhances the understanding of DFP as a co-substrate for optimizing methane recovery from AD of CM.

## Simple Summary

The traditional perspective of waste disposal is no longer appropriate, as the circular economy recognizes organic waste as a key potential for both nutrition and energy recovery. Anaerobic digestion has proved high efficacy as a sustainable technique for acquiring energy from organic wastes with fewer adverse environmental effects. Chicken manure rich in organic matter is a significant agricultural pollutant associated with greenhouse gas emissions and water pollution. Due to the low carbon-nitrogen ratio of chicken manure, acidification could quickly occur in the anaerobic digestion process, reducing anaerobic digestion efficiency. Therefore, the application of chicken manure for anaerobic digestion has become a hot topic. The study was designed to improve the biogas/methane production and enhance the core microbial interaction of anaerobic digestion. The addition of dragon fruit peel as a co-substrate was suggested to determine the optimal ratio that reduces the low carbon nitrogen ratio and improves methane potential. The co-substrate dragon fruit peel at an optimal ratio maximizes the biogas/methane yields up to 411.1 and 180.2 mL/g VS, respectively. The study findings contribute to a deeper understanding and the approach of dragon fruit peel as a co-substrate for methane recovery from anaerobic digestion of chicken manure.

## 1. Introduction

Given the issues of global warming and the swift rise in energy demand, there is a growing focus on generating renewable energy from organic waste [1]. China ranks as one of the largest chicken farming countries globally, producing approximately 9 billion chickens each year [2]. As a result, approximately 155 million tons of chicken manure (CM) are generated annually [3,4]. Organic matter and nutrient-rich CM are major agricultural pollutants linked to greenhouse gas emissions and water contamination [5]. Treatments and the recycling of CM must occur before disposal, due to its high volatile solid (VS) content, ranging from 60% to 85%, and its rich biodegradable organic matter [6,7]. The main methods for managing CM are aerobic composting, high-temperature drying, and anaerobic digestion.

Aerobic composting is a well-established technique that necessitates substantial energy input. The process converts degradable organic materials in CM into CO_2_, potentially causing nitrogen loss, air pollution, and increased greenhouse gases [5,8]. High-temperature drying is an efficient method; however, it entails considerable energy consumption, contributes to air pollution, and results in diminished product quality. In contrast, anaerobic digestion (AD) effectively converts degradable organic materials into clean energy, reducing nitrogen loss and supporting the United Nations’ sustainable development goals [9,10]. The effectiveness of AD mainly relies on the properties of the substrates, the operational parameters and conditions, and the diversity of the microbial communities involved in the process [11]. Diverse microbial communities are crucial players in the methanogenesis process that occurs during anaerobic digestion. Fermentative and syntrophic acetogenic bacteria, along with methanogenic archaea, collaboratively enhance the efficiency of the AD process [12]. Microorganisms are vital for breaking down complex molecules into simpler substances across fermentative, acidogenic, acetogenic, and methanogenic stages [13,14]. Because of the CM’s low carbon-to-nitrogen ratio (C/N) from 3:1 to 13:1, acidification can rapidly happen in the AD process alone, leading to decreased efficiency of the AD reactions [10]. A 5–10 times increase in methane production was observed by Wang et al. (2020) [15], attributed to the addition of allophane, which mitigated the inhibition associated with the digestion of CM. This inhibition was mainly caused by high concentrations of ammonia nitrogen [15]. The primary factor is that elevated levels of nitrogen considerably hinder the AD process, primarily affecting methanogenesis [16,17]. Furthermore, high ammonia nitrogen disrupts methanogens, particularly acetoclastic types, and shifts microbial metabolism towards syntrophic acetate oxidation under nitrogen stress [18,19]. Gradual increase in ammonia in the reactors may develop resilience, for instance, acetate oxidation, and favor tolerant hydrogenotrophic species [20]. Consequently, co-digestion with carbon-rich substrates can be used to buffer ammonia toxicity to sustain methane production from CM digestion [21,22]. As a result, the effective use of CM in AD has emerged as a prominent area of research.

Researchers have investigated multiple approaches to alleviate the inhibitory impact of ammonia during AD. These techniques include anaerobic co-digestion (AcD), membrane distillation, stripping, pH and temperature control, and concentration buffering to reduce ammonia nitrogen levels. The AcD process, which employs various substrates, holds promise in overcoming the constraints linked to the mono-digestion of a singular feedstock. This methodology improves digestibility by leveraging substrate interactions, enhances process stability, boosts biogas yield, promotes microbial diversity, and increases digestate nutrition [23,24]. Wang et al. (2022) [25] investigated the mixing ratio of excess sludge with CM, and an increased methane yield of 82.4–123.1 mL/g VS at mesophilic and 33.9–171.3 mL/g VS at thermophilic conditions was recorded. The results showed that co-digesting excess sludge and CM at a ratio of 1:1.5–2 can produce high methane and good digestate dewaterability [25]. Selecting suitable co-substrates is vital for efficient AD, as properties like C/N ratio, biodegradability, and nutrients affect microbial activity, methane production, and stability [26]. Fruit biowaste represents a significant byproduct of advancements in food production. According to the Food and Agriculture Organization (FAO), about 1.3 billion tons of edible food are wasted each year, which adds to environmental harm and uses up valuable resources [27]. The byproduct percentage may make up roughly 60% of the fruit [28].

Dragon fruit (DF) belongs to the Cactaceae family and has two varieties typically differentiated by their pulp colors, i.e., red pulp (*H. polyrhizus*) and white pulp (*H. undatus*) [29]. The DF is a tropical fruit, and its peel constitutes over 25% of the total fresh fruit weight.This by-product is usually discarded as waste during fruit processing [30]. Discarding these peels as agricultural waste is common, raising environmental issues and resulting in inefficient resource use [31]. The waste from DF causes numerous problems, including disease, air, and water pollution to the environment, especially since most of this fruit waste contains a lot of water that is very prone to rot [31]. This waste has been used for decades, mainly as animal feed, and its antimicrobial and antioxidant properties have been studied. Aside from that, the peel has limited uses and is regarded as waste, often discarded in landfills. Its potential as an alternative energy source (biogas, bioethanol, or biohydrogen) has been explored in some cases [32,33,34]. However, the chemical compositional characterization includes 72% carbohydrates with ~47% dietary fibers of dry weight and minimal soluble sugars [35,36]. Its lignocellulose components follow a distinct ratio of cellulose > hemicellulose > lignin, which enhances biodegradability [35,37]. Previous studies suggest that the anaerobic co-digestion of CM in conjunction with carbon-rich agricultural residues can create a well-balanced nutrient system. This encompasses refined carbon-to-nitrogen ratios, ideal pH levels, augmented buffer capacity, system stability, and heightened methane production [4]. Higher carbon content accelerates fermentation, including hydrolysis and acidogenesis, which results in reduced methane yield [38]. While carbon-rich fruit waste can enhance methane production under the right conditions, it is crucial to carefully manage the AD to prevent transient acidogenesis and the associated challenges of VFA accumulation. The AD can be improved through thoughtful co-digestion and operational strategies that optimize microbial health and ward off the risks of digester souring [39]. Nitrogen is a supplementary major nutrient and could be used for C/N adjustment to the optimum range of 20–30 [25]. Zhao et al. (2024) reiterate that co-digestion not only improved yield but also facilitated greater interaction between bacteria and methanogenic archaea [40].

The literature review revealed that many studies have investigated the AcD of food and agricultural waste with co-substrates (CM), and a few have focused on fruit waste. Likewise, the microbial and biochemical pathways in AD have also been well documented; however, a key applied challenge remains in efficiently digesting nitrogen-rich waste (CM) without process inhibition. Despite extensive research on CM co-digestion with agricultural wastes, critical gaps persist, such as the lack of quantitative data on DFP availability and its comparative performance as a co-substrate relative to other well-studied wastes, an insufficient understanding of microbial community shifts, and unvalidated hypotheses regarding the optimal DFP-CM ratio to balance the C/N ratio to enhance methane production. Consequently, this study aimed to address this challenge by utilizing a strategic carbon-rich co-substrate DFP to mitigate high ammonia by the optimization of the C/N ratio and potential for biogas/methane production from the mono- and co-digestion with CM. This is the first attempt to systematically explore mesophilic AcD of DFP and CM coupled with the interpretation of microbial shifts linked to process performance. Additionally, an effort was made to demonstrate the successful optimization of an AcD formula using a specific, underutilized fruit waste to achieve reliable bioenergy recovery, which can offer a direct pathway for practical application in agricultural waste management.

## 2. Materials and Methods

### 2.1. Substrate Collection and Characterization

The CM was collected from poultry facilities and screened to remove large solid particles. The DF was sourced from a local fruit market. The peel was manually separated, then dried at 80 °C until a constant weight was achieved. Drying at this temperature can effectively reduce moisture content while preserving the critical components (essential carbohydrates) necessary for subsequent digestion processes, while improving biomass solubility and enhancing microbial availability during anaerobic digestion [41,42,43]. The dried DFP was ground-shredded by a high-speed mixer grinder (XueErHui, Wellybo Electric Appliance Co., Ltd., Zhongshan, Guangdong, China) and sieved through a 3 mm mesh, and a uniform 3 mm particle size was achieved. The recommended particle sizes range from 0.1 mm to 30 mm for optimal biomethane production during AD [44]. The gathered CM and DFP were conveyed to the laboratory, securely sealed in an airtight plastic zipper bag, and maintained at 4 °C for subsequent physicochemical analysis and AD experiments. Samples were dried for 24 h at 105 °C and subsequently incinerated for 4 h at 550 °C to determine the biomass’s moisture content, ash content, total solids (TSs), and VS [45]. An elemental analyzer was used to determine the total amounts of carbon, nitrogen, hydrogen, and sulphur (Elementar analyzer system GmbH, Langenselbold, Hesse, Germany). The anaerobic digested sludge (ADS) utilized in the present mono- and co-digestion as an inoculum source was obtained from a large-scale operational anaerobic biogas facility. The pH of CM and ADS was assessed right after collection, prior to their transport to the laboratory. The pH values in the anaerobic reactors were tracked after every seven days during the incubation period using a pH meter (PHS-3C, YOKE Co., Beijing, China). The ADS was maintained at 37 °C for about 10 days to facilitate the decomposition of the available organic matter, thus reducing the potential impact of biogas generated by the ADS on the experimental outcomes.

### 2.2. Batch Mono- and Co-Digestion Experimental Set-Up

The batch mono- and co-digestions of CM and DFP were conducted in 500 mL glass anaerobic reactors with a 350 mL working volume with 150 mL headspace to find the optimal mixing ratio for the highest methane production yield. Five treatment groups were run in triplicate for 25 days, focusing on the mono-digestion of CM and DFP, as well as the co-digestion of CM and DFP at 25:75, 50:50, and 75:25 g/g ratios (on a C/N basis). A total of 120 mL of degassed ADS was added to all anaerobic reactors. The reactors contained CM and DFP mono-digestion mixtures with a VS based substrate-to-inoculum (S/I) ratioof 0.7–1.4 and co-digestion mixtures with an S/I ratio of 0.8–1.2. Subsequently, the reactors were purged with 99.9% nitrogen gas for 15 min to ensure anaerobic conditions (Table S1). Starting an anaerobic digestion system is a delicate and crucial step for successful operation. To perform this, a specific amount of inoculum was added to the digester along with the substrate, supplying essential microorganisms to kick-start the digestion process. The biodegradation rate, lag time, and potential substrate degradation depend on the concentration of microorganisms [46]. The anaerobic reactors were maintained at 37 ± 1 °C in a shaking incubator operating at 120 rpm to facilitate continuous shaking and ensure optimal substrate mass transfer and microbial interaction without causing excessive shear stress and biomass washout. The biogas produced was collected in a 250 mL aluminum Tedlar bag (LB-301 Delin, Dalian, China), connected to the AD reactors through a stainless-steel rubber valve. The daily biogas volume during AD was quantified using a 100 mL syringe. A modified kinetic Gompertz Model (Equation (1)) was used to illustrate how different concentrations of CM and DFP influence the kinetic parameters of the AD system. After the completion of the experiment on day 25 of the incubation period, the digested feedstock liquid samples were collected for microbial analysis.

(1) $$M=M_{max}\times \exp\left\{-\exp\left(\frac{R_{m}\times e}{M_{max}}\left(\lambda -1\right)+1\right)\right\}$$

Here, M_max_ represents the amount of biogas/methane mL/g VS produced at a given time t, M represents the total amount of biogas/methane produced mL/g VS, λ represents the log phase (d), R_m_ is the rate of maximum biogas/methane produced mL/g VS (d), t is the time period (d), and e is equal to 2.718. The statistical analysis for biogas and methane production was performed by using a paired Student *t*-test (*p* < 0.05), which was conducted using the GraphPad Prism (10.4.1) Software, Inc., San Diego, CA, USA.

### 2.3. Analytical Methods

The composition of biogas was examined using the GC-2200 (Yunbo Instruments, Chengdu, China), which is outfitted with a thermal conductivity detector and a 2 m TDX-01 column. The injector, oven, and detector operating temperatures were 150, 100, and 150 °C, respectively. A total of 500 µL of the biogas sample was loaded into the GC using a gas-tight syringe, and argon was used as the carrier gas, flowing at a rate of 0.2 mL min^−1^ [47].

The digestate liquid samples for the VFA analysis were collected every seven days from the anaerobic mono-and co-digesters of CM and DFP. The VFAs were quantified in the supernatant after 2 mL of the digestate sludge sample was centrifuged (HR-26, Benchtop high speed, Zhengzhou, China) at 2500× *g* for 10 min. After adding 0.2 mL of H_3_PO_4_ to 1 mL of the supernatant, the mixture was centrifuged at 9000× *g* for two minutes after being kept in an ice bath for thirty minutes to examine the VFAs. The supernatant was filtered using a syringe filter with a 0.22 μm pore size before being put into GC vials. The VFAs were analyzed using gas chromatography (GC-2200, Yunbo Instruments, Chengdu, China) with an FID detector and a DB-FFAP column (30 m 0.0.25 mm 0.25 m; Agilent Technologies, Inc., Santa Clara, CA, USA). Argon was used as the carrier gas at a flow rate of 0.2 mL min^−1^, the detection time for the VFAs was set 17 min, and the injector, oven, and detector were kept at 240, 130, and 240 °C, respectively [47,48]. The GC was calibrated before analyzing the digestate VFA samples, adjusting the pressure and checking the carrier gas flow rate, and comparing the setpoint to observed flows at different rates. For the liquid, solutions were prepared from an aqueous stock solution of 2500 mg/L that contained eight organic acid compounds, such as acetic (C2), propionic (C3), iso-butyric (iC4), n-butyric (nC4), iso-valeric (iC5), n-valeric (nC5), iso-caproic (iC6), and n-caproic (nC6), by gravimetrically weighing the individual components’ purity.

### 2.4. Microbial Community Analysis

The liquid samples obtained from each anaerobic reactor after an incubation period of 25 days were utilized to analyze the arrangement of the microbial community via high-throughput sequencing (16S rRNA) [49,50]. EZNA^®^ soil DNA Kits (Omega Bio-Tek, Norcross, GA, USA) were used to extract total genomic DNA in accordance with the manufacturer’s instructions. The extracted DNA was examined on a 1% agarose gel, and its concentration and purity were assessed with the NanoDrop 2000 UV–vis spectrophotometer (Thermo Scientific, Wilmington, NC, USA). To analyze microbial biodiversity, the V3–V4 variable region of the 16S rRNA gene was targeted and amplified using an ABI GeneAmp^®^ 9700 PCR thermocycler (ABI, Foster, CA, USA). Separate primers (806R 5-GACTACHVGGGTATCTAATCC-3 and 519F 5-CCTACGGGNGGCWGCAG-3) and archaeal primers (Arch-915R 5-GTGCTCCCCCGCCAATTCCT-3 and Arch-349F 5-GYGCASCAGKCGMGAAW-3) were used for bacterial and archaeal 16S PCR amplification. The PCR products were run on a 2% agarose gel, then extracted and purified with an AxyPrep DNA Gel Extraction Kit (Axygen Biosciences, Union City, CA, USA) following the manufacturer’s instructions. Subsequently, DNA was quantified using a Quantus™ Fluorometer (Promega Corp., Madison, WI, USA).

Amplicons, after purification, were pooled in paired-end and equimolar sequenced (2 × 300) as per standard procedures (Majorbio Bio-Pharm Technology Co., Ltd. (Shanghai, China)), on an Illumina MiSeq platform (Illumina, San Diego, CA, USA). The raw 16S rRNA gene sequencing reads were de-multiplexed, then subjected to Trimmomatic to filter the quality, and merged by FLASH. To cluster the operational taxonomic units (OTUs) with a 97% similarity cutoff, UPARSE (version 7.1, http://drive5.com/uparse/) was used, and chimeric sequences were identified and removed. For taxonomic analysis, the RDP Classifier (http://rdp.cme.msu.edu/) was used to analyze each OTU representative sequence against the 16S rRNA database based on a 0.7 confidence threshold.

### 2.5. Statistical Analysis

The α-diversity was assessed by OTU numbers and Chao 1, Simpson, and Shannon diversity indexes, with phylogenetic distance indicated. For β-diversity compression of microbes among the samples subjected to different CM and DFP loading levels, a principal coordinates analysis (PCoA) of unweighted UniFrac distances was carried out using the Bray–Curtis similarity index in PAST v3. Each experiment included three biological replicates, presenting findings as mean ± standard deviation (SD). Using the SPSS (IBM Corp., v. 20.0, Armonk, NY, USA), we conducted one-way ANOVA and least significant difference (LSD) tests to evaluate significant differences at *p* < 0.05.

## 3. Results and Discussion

### 3.1. Characterization of the Substrates

Physicochemical analysis of the substrate and ADS showed that ADS has the lowest total solid (TS) content (6.4 wt.%) and VS content (4.1 wt.%), and a higher moisture content (93.6%) than DFP (44.9%) and CM (68.2%). The highest TS of 56.1% was reported for CM, while a slightly lower TS of 35.8% was calculated for DFP, respectively (Table 1). The high levels of biodegradable components, such as VS and moisture content, are beneficial for efficient AD [49]. The DFP showed comparatively higher C/N ratios (40.3) than those of CM (13.2) (Table 1). Since CM has a higher nitrogen content (3.6), the co-substrate DFP, with a higher carbon content, addresses this by maintaining the C/N ratio. Previous research shows that anaerobic co-digestion of CM with carbon-rich agricultural waste balances nutrients and system conditions. It improves C/N ratios, pH, buffer capacity, system stability, and methane yield [50]. Excess free ammonia, originating from the AD of nitrogenous organic matter, significantly impacts digestion stability by inhibiting AD [51]. Low C/N ratios result in ammonia buildup, which negatively affects methanogenic archaea [49]. Although ammonia toxicity is a recognized problem, the underlying mechanism is not well understood, and determining a ‘critical’ threshold concentration appears difficult, with reported values ranging from 1.5 to 7 g/L [52]. Anaerobic co-digestion can alleviate ammonia inhibition by increasing the C/N ratio of substrates [6].

### 3.2. Biogas and Methane Generation

**Figure 1:**
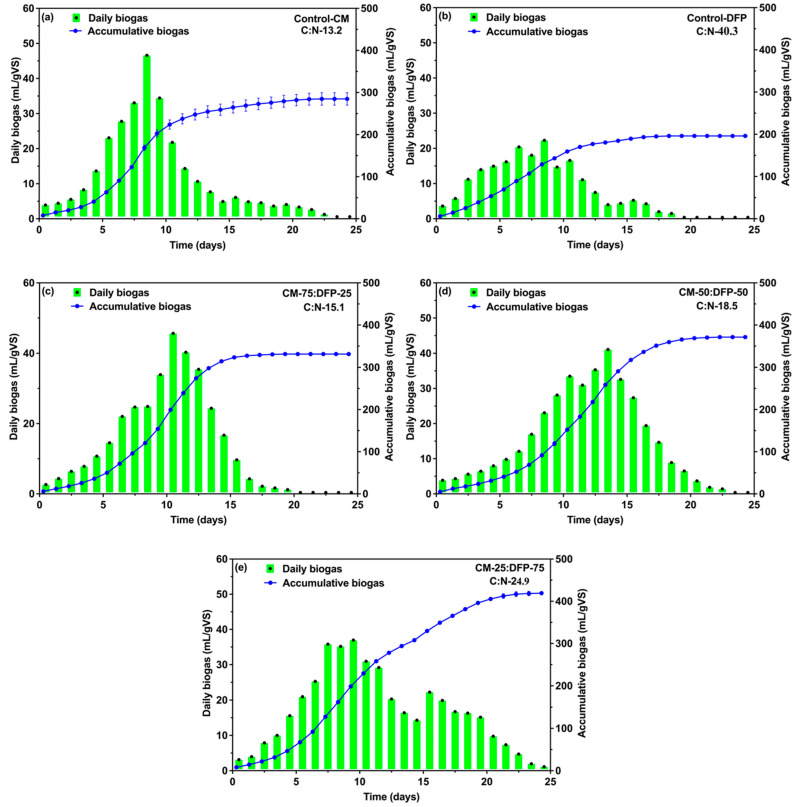
Daily and accumulative biogas production patterns for control mono-digestion of chicken manure (CM) and dragon fruit peel (**a**,**b**) and CM and co-digestion (**c**–**e**).

The biogas produced during each trial was crucial for evaluating the feasibility of the AD process. Figure 1 illustrates the daily and cumulative biogas production for each treatment throughout the 25-day incubation period. The daily biogas yield from controls and different ratios of CM and DFP, such as CM-75:DFP-25, CM-50:DFP-50, and CM-25:DFP-75 under control conditions, was low at the initial phase. Subsequently, high biogas production peaks of control CM and DFP were observed from days 6–11, and the highest was recorded as 46 mL/gVS/d and 21.9 mL/gVS/d, respectively (Figure 1a,b). In the CM mono-digestion reactor, the daily biogas volume decreased sharply from day 11 onward and then remained stable for remainder of the 25-day incubation period, while the daily biogas production from DFP mono-digestion reactor exhibited a steady trend. Daily biogas production peaked during the early digestion phase (days 6–11), with values of 45.5, 33.1, and 37 mL/gVS/d recorded for the CM-75:DFP-25, CM-50:DFP-50, and CM-25:DFP-75 co-digesters, respectively (Figure 1c–e). The generated biogas increased until it reached a peak, then declined, and was followed by several additional peaks as the digestion continued. A previous study of two substrates, banana pseudo-stems and CM in AcD, resulted in 57.2 and 66.1% more methane as compared to the control reactor [26,53]. Anaerobic co-digestion is the synergistic digestion of two or more biodegradable substrates, which results in higher efficiency and productivity than the digestion of a single substrate [54]. At mesophilic temperatures, the microorganisms present in the co-substrates significantly affect the hydrolysis rate [55]. Dynamic simulations were used to validate the synergistic impact of co-substrate addition on sludge methanization, and an increase in hydrolysis rate from 1.5 d^−1^ to 2.5 d^−1^ was identified for simulating the measured gas production rate. Wang et al. (2012) [56] used a mixture of dairy manure, CM, and wheat straw based on optimized feeding and a 25:1 C/N ratio, within the required range for proper digestion. All had significantly higher biogas production than CM, dairy manure, and wheat straw alone. In addition, co-digestions where dairy manure or CM was mixed with WS also showed higher biogas potential, as well as VS removal rates, than for the digestion of dairy manure or CM alone [56]. CM exhibits strong hydrolysis capabilities when co-digested with other organic waste; however, the excessively high TS of CM (56.1%) in the co-substrate may lead to poor mass transfer [57]. Biogas yields rose as the C/N ratio increased because total organic carbon serves as the primary substrate for carbonaceous biogas [58,59]. The integration of diverse substrates improves operational parameters such as the C/N ratio and fosters mutualism among microorganisms by enriching both trace and macronutrients [60].

Under anaerobic mono- and co-digestion of CM with DFP, the relative accumulative biogas generation volumes were assessed over 25 days (Figure 1a–e). The highest accumulative biogas yield of 411.2 mL/gVS was obtained when CM was co-digested with DFP at a 25:75 ratio. This was followed by yields of 366.4 mL/gVS at a CM-50:DFP-50 mix and 325.5 mL/gVS at a CM-75:DFP-25 ratio. In contrast, the mono-digestion of CM produced 276.8 mL/gVS and DFP 190.1 mL/gVS. Co-digestion with diverse co-substrates is frequently employed to sustain significant waste streams. Including different organic industrial waste through co-digestion leads to a notable increase in biogas production [61]. The anaerobic biodegradability of grass was enhanced through co-digestion with CM at a 20:80 VS ratio and 21.70 C/N ratio, and the accumulative biogas yield was the highest, at 237 mL/gVS. The enhancement of biogas production was attributed to the buffering effects of ammonia and rich trace elements in CM. Combining animal manure with a low C:N ratio and feedstock with a high C:N ratio and low nitrogen improved process efficiency and methane yield compared to manure alone.

**Figure 2:**
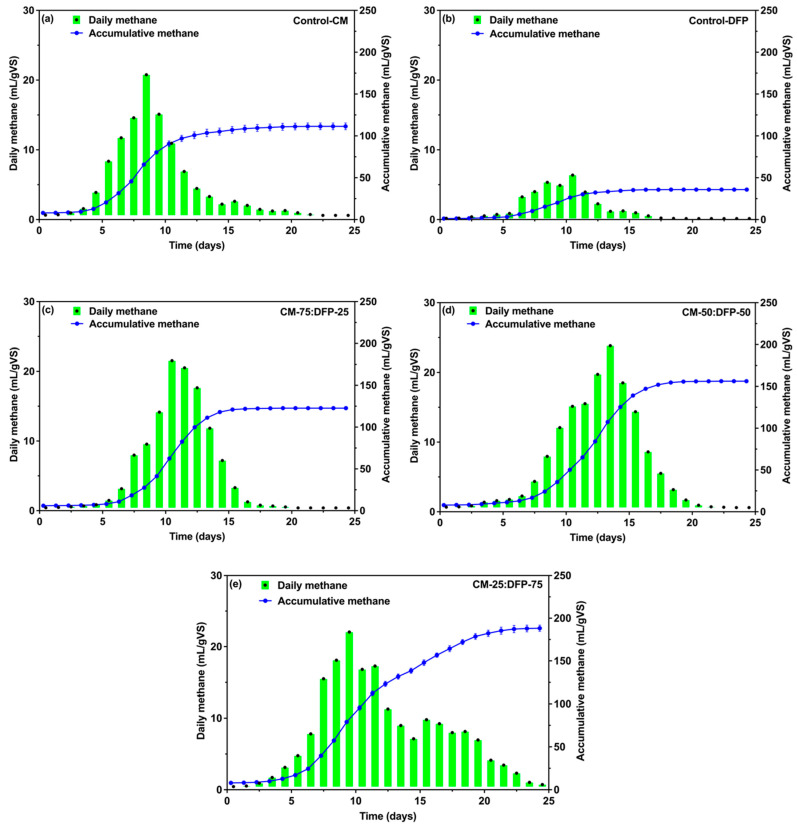
Daily and accumulative methane yield patterns for control mono-digestion of chicken manure (CM) and dragon fruit peel (DFP) (**a**,**b**) and co-digestion (**c**–**e**).

The stability of an anaerobic system could also be determined by the amount of methane produced. The amount of methane generated often serves as an indicator of the digester’s operating effectiveness. Methane serves as the terminal metabolite of AD and can directly indicate the metabolic activity of methanogens [62]. The daily methane production of the different experimental groups showed two to three peaks during the AD period (Figure 2a–e). The highest peak values of CM and DFP co-digestion at 75:25 were 21.7, 23.2 at 50:50, and 21.1 mL/g VS at 25:75, respectively (Figure 2c–e). The control groups were 20.2 at CM and 6.2 mL/g VS at DFP (Figure 2a,b). The daily methane trends during the induction period in the co-digestion of the CM and DFP groups were notably similar, possibly due to the C/N ratio of (24.9) at CM-25:DFP-75, (18.5) at CM-50:DFP-05, and (15.1) at CM-75:DFP-25 (Table S1). In AD systems, the ratio of C/N in substrates substantially affects the growth and metabolic processes of microorganisms [63]. Previous studies have shown that the inhibition or toxicity associated with nitrogen-rich substrates is mainly due to the accumulation of ammonia in the liquid phase of the anaerobic digestate [64]. Zheng et al. (2021) reported that methane production remained low in an ammonia-inhibited reactor fed with low C/N substrates, indicating that the system was operating under an “inhibited steady state” [65]. Therefore, incorporating an appropriate amount of DFP into the CM significantly boosted methane production from the CM.

Notably, the highest cumulative methane production was observed after adding an appropriate ratio of co-substrate DFP, as the accumulative methane production curve of CM-25:DFP-75 was much higher than that of the other groups (Figure 2e). The higher potential for methane production from CM-75:DFP-25 was 116.7, 148.3 from CM-50:DFP-50, and from CM-25:DFP-75 it was 180.3 mL/g VS, respectively (Figure 2c–e). This shows that incorporating DFP substantially enhanced the methane yield potential. Regression analysis using the Modified Gompertz Model was well-fitted with experimental biogas/methane, having a correlation coefficient (R_2_) between 0.997 and 0.998 (Table 2). This model was used to fit the cumulative biogas/methane production, and the obtained parameters included the final biogas/methane production Mmax (275, 194, 337, 392, and 433) and (36–181), maximum biogas/methane production rate Rmax biogas (0.008e–0.864e) and methane (7.545e–0.001e), and lag phase (λ). The results indicated that the predicted final methane production was in close agreement with the actual measured values (Table 2). The predicted final methane production closely matched the actual measured values, confirming the model’s reliability. The lag phase parameter (λ) represents the initial period before microorganisms adapt to the new environment, proliferate, and enter the exponential growth phase. Generally, a larger λ indicates slower microbial adaptation and delayed enzymatic activity, resulting in a slower onset of methane production [66]. Comparing λ values among treatments during biogas/methane production, CM exhibited a lag phase of 0.347 and 0.559 days, while CM-75:DFP-25 exhibited 0.316 and 0.469 days, CM-50:DFP-50 0.242 and 0.369 days, and CM-25:DFP-75 showed a shorter lag phase of 0.196 and 0.241 days, suggesting that CM-DFP co-digestion treatments accelerated microbial adaptation and enzyme activation, with the most pronounced effect observed in the CM-25:DFP-75 treatment. Zahan et al. (2018) [50] studied AD of four agro-industrial wastes including CM, food waste, wheat straw, and hay grass, at six different ratios, with all mixtures having a C/N ratio of 20 or more under the semi-continuous operational conditions. Notably, increases in biogas production of 73.0, 167.2, and 116.9% were observed at the optimum substrate mixture of CL:FW:WS 60:20:20 in comparison to biogas production of CM mono-digestion [50]. In the process of co-digesting maize straw and CM at a total solid (TS) concentration of 5%, a notable synergistic effect was observed, resulting in methane production of 218.8 mL/gVS at a 1:1 ratio [67]. Likewise, the co-digestion of CM and *Chlorella* sp. at various ratios (0:10, 2:8, 4:6, 6:4, 8:2, 10:0) and the highest methane yield of 169.7 mL/gVS was achieved at an 8:2 ratio compared with 94 of *Chlorella* sp. and CM 146.2 mL/gVS mono-digestion. Mono-digestion of manure often results in low methane production and extended retention times due to a lack of adequate macro- and micronutrients, an imbalanced C/N ratio, and unfavorable organic loading rates [62]. Co-digestion may have synergistic effects that improve methane production characteristics or raise the specific methane output of the particular substrate [68]. The synergistic effect frequently relied heavily on the composition of the biomass mixture undergoing co-digestion [1]. Yangin-Gomec and Ozturk (2013) found that the daily biomethane production of cow and chicken waste increased by 1.2 times when mixed with corn feed [69]. Thus, based on the results obtained during methane production assays, co-digestions of CM and DFP with ratios of 25:75 were recommended due to their stable AD performance and the highest energy recovery.

### 3.3. Stability Parameters of Digesters

**Figure 3:**
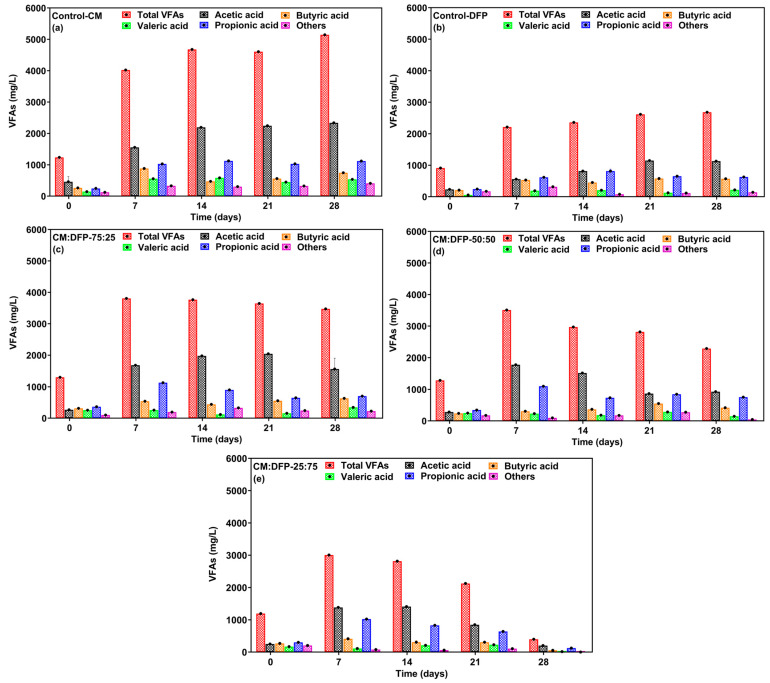
Volatile fatty acid (VFA) production trend and conversion at chicken manure (CM) and dragon fruit peel (DFP) mono-digestion (**a**,**b**) and co-digestion (**c**–**e**).

To examine how the co-substrate DFP influences the digestion performance of CM under mesophilic conditions, the digestion performance for each group was assessed, and the concentration of VFAs and pH were compared. Digestion performance of the CM-25:DFP-75-treated group was superior in terms of biogas/methane yield to that of the control-CM and other co-digesters groups. VFAs are products of acidogenesis, and their composition and quantity depend on operational parameters, substrate composition, and the available microbial community [70]. Acetic, propionic, and butyric acids play crucial roles in the intermediate stages of AD. Acetic acid is a precursor for methane production and supports methanogenic microorganisms, while propionic and butyric acids contribute to microbial community balance [13]. In the entire incubation period, the VFA production of control-CM and co-digesters at 75 and 50 CM was slightly higher than that of CM-25:DFP-75 and was observed to accumulate in the reactors (Figure 3). The appropriate C/N and the optimal ratio of co-substrate DFP at co-digestion CM-25:DFP-75 improved the buffer capacity, significantly controlled VFA production, and achieved better VFA degradation. Interestingly, a significant amount of VFAs were detected in the control CM and other co-reactors having CM and DFP compared to CM-25:DFP-75 at the end of the incubation period. The concentration of VFAs in CM was recorded at the beginning as the lowest, 1236.31 mg L^−1^, and the highest was 4677.1 mg L^−1^ at day 14, which shows the partial conversion of the produced VFAs. While a stable VFA production of 1193.3–3006.5 mg L^−1^ at CM-25:DFP-75 was recorded, the continuous conversion of these VFAs validated a high methane yield (Figure 3e). The inhibitory effect of VFAs on the production of biogas and also on the methane-to-carbon-dioxide ratio is evident above 6 g L^−1^ [71]. Similarly, the VFA concentration of CM and DFP co-digesters at ratios 75:25 and 50:50 was higher than that of CM-25:DFP-75 but lower than that of the CM control, as an excessive accumulation of VFAs was observed. This imbalance impedes the AD process, as methanogenic archaea are particularly susceptible to acidic conditions, which can inhibit or halt their growth [1]. A study showed that the valorization of reground pasta showed a conversion yield into fermentation products (FPs) of 54 ± 2% in terms of Chemical Oxygen Demand (CODFP/totCODin) was obtained with a concentration of 18.1 ± 1.0 g CODVFAs/L composed by 69% odd (6.5; 3.8; 2.7 gCOD/L of acetic, iso-butyric and butyric acids, respectively) and 26% even (4.0 and 1.0 gCOD/L of propionic and valeric acids, respectively) [72]. While constant consumption demonstrates the reactor stability, the accumulation of VFAs indicated the kinetic uncoupling between acid-producing and acid-consuming microbes within the reactor [73]. The results indicate that the reactors supplemented with the co-substrate DFP exhibited superior performance in comparison to the control-CM and the co-digesters with elevated concentrations of CM.

The principal acids generated throughout the acidogenesis process in this investigation are illustrated in Figure 3. The initial concentration of acetic acid in the digesters was below 0.500 mg L^−1^. Throughout the 25 days, acetic acid and propionic acid were identified as the primary VFAs across all digesters, with noted increases in the concentrations of both acids. However, their concentrations were significantly reduced in the CM-25:DFP-75 co-digester, compared with the CM and DFP mono-digestion controls. In contrast, only minimal increases and consumption of thes acids were observed in CM-75:DFP-25 and CM-50:DFP-50 co-digesters (Figure 3a–e). High concentrations of VFAs can inhibit methanogenic microorganisms, resulting in lower conversion of VFAs to methane. This can lead to VFA concentrations further acidifying the medium and lowering the pH [52,74]. The control-CM demonstrated the highest ratios of acetic-acid-to-propionic-acid, as well as the greatest yield of VFAs at 75 and 50. The optimal CM:DFP ratio for VFA production and conversion to methane was determined to be 25:75.

The temporal dynamics and interrelationships of key factors affecting AD stability, including pH, alkalinity, VFAs, and free ammonia, as well as their impacts on methane production profiles, were further investigated. Regarding the behavior of pH over time, it was observed that the pH of all digesters was between 7.4 and 7.8 (Figure S1). According to the literature, methanogenesis is permanently inhibited at pH values lower than 5.5 [75,76,77]. However, pH levels varied with VFA concentrations. The accumulation of VFAs generally leads to a reduction in pH. The VFAs produced from CM consisted mainly of acetic acid and propionic acid. As the digestion proceeded, the composition of acetate decreased gradually as the conversion of VFAs to methane was more than that of production. Excessive VFAs could then disrupt the balance of the digestion process, thereby inhibiting methanogens [78]. No significant difference in the pH of the co-digester CM-25:DFP-75 was observed, while a notable drop in pH in the control-CM and a slight decrease in the co-digesters at 75 and 50 ratios were recorded. The daily and cumulative methane yield of the present study with the addition of 25% DFP is shown in Figure 2e. From the figure, it can be seen that the methane potential was recorded as the highest with the addition of DFP. The methane peak value for the CM-25:DFP-75 mixture was 180.3 mL/g VS, compared with 100% CM, DFP, and other co-digested treatment groups. The addition of appropriate DFP creates an alkaline environment that helps maintain the nutrient and pH balance. In anaerobic digestion, the optimal pH should be kept between 6.8 and 8.0 [79]. The variation in biogas production may result from changes in microbial metabolism, influenced by pH fluctuations in the digestate [80]. This pattern was noted in a prior study regarding the AD of CM with lemongrass [80]. In addition, manures that have low C/N ratios contain relatively high concentrations of ammonia, exceeding that necessary for microbial growth and probably inhibiting AD [56].

### 3.4. Structure of Microbial Community

**Figure 4:**
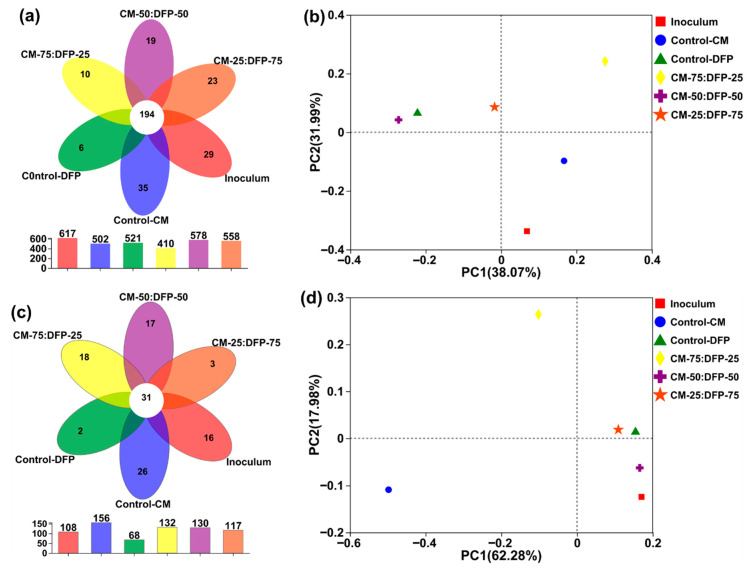
Unique and shared OTUs of the mono-digestion and co-digestion of chicken manure (CM) and dragon fruit peel (DFP) bacteria (**a**) and archaea (**c**) are shown in Venn diagrams. Weighted assessment of microbial community distances (**b**) bacteria and (**d**) archaea using principal coordinate analysis (PCoA).

To investigate microbial responses in anaerobic mono- and co-digesters with CM and DFP, the microbial community of ADS (inoculum) from a large-scale biogas facility was used as a reference to measure community changes. Figure 4b,d shows a weighted assessment of microbial community distances and ranking. The alterations in bacterial and archaeal communities represented 38.1% and 62.3% of the total shifts along the PC1 axis, respectively. A notable similarity appears in the co-digesters CM-50:DFP-50, CM-25:DFP-75, and control-DFP versus control-CM and CM-75:DFP-25. This indicates that DFP, as a co-substrate, could lessen CM inhibitory effects and alter the microbial community structure. The effectiveness of AD is significantly affected by the diversity and distribution of microbial communities, since imbalances within these functional groups may lead to process failure [13].

#### 3.4.1. Bacterial Community

The distribution of OTUs in the Venn diagrams (Figure 4a,c) was generated to recognize the alterations in the overall and taxa-specific OTUs after adding the co-substrate DFP. The common OTUs shared among the control and treated groups CM and DFP (75:25, 50:50, and 25:75%) were 194, while the specific OTUs were the highest for the treatment group CM-50:DFP-50, followed by 25:DFP-75 (Figure 4a). Using various concentrations of DFP resulted in the highest OTU counts, and slight variations were also noted among the treated groups.

**Figure 5:**
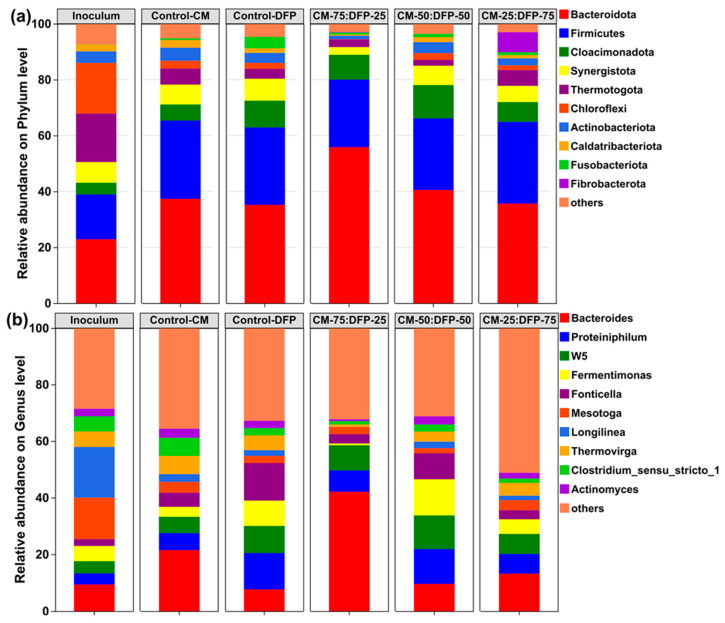
Bacterial community composition of inoculum, and mono- and co-digestion of chicken manure (CM) and dragon fruit peel (DFP) at the phylum (**a**) and genus levels (**b**).

An investigation of the microbial community was conducted at the phylum level to elucidate the alterations in functional microorganisms across various treatments. The dominant phyla, including *Bacteroidetes*, *Bacillota*, *Cloacimonadota*, *Synergistota*, *Fibrobacterota*, and *Thermotogota*, accounted for 84–88% of the total sequence reads (Figure 5a). *Bacillota* generate extracellular enzymes like cellulase, lipase, and protease, which mainly support cellulose metabolism, proteins, lignin, and lipids, and are strongly linked to substrate hydrolysis [81]. This indicates that a significant quantity of organic material is present for degradation in the substrates. The relative abundance of *Bacteroidetes* and *Bacillota* in the treatment groups at CM-75:DFP-25, CM-50:DFP-50, and CM-25:DFP-75 was higher as compared to the control reactors. *Bacillota* can perform a critical function in producing VFAs, substantially contributing to the increase in methane production. Interestingly, the enrichment of *Fibrobacterota* (0–7.2%) was observed in the treatment group CM-25:DFP-75, which was completely missing in all treatment groups except CM-25:DFP-75 (Figure 5a). The phylum *Fibrobacterota* is primarily found in mesophilic anaerobic environments. Xu et al. (2021) indicate that *Fibrobacterota* may play an active role in fiber digestion [82]. *Fibrobacteria* contain cellulase in their periplasm, allowing for the decomposition of cellulose for microbial uptake [83]. *Fibrobacterota* is known to contribute to the degradation of cellulose and hemicellulose, having genes that can produce cellulase and xylanase [84]. Lv et al. (2019) reported that in anaerobic digesters fed with chicken manure and maize silage, high ammonia concentrations resulted in a drastic reduction in *Fibrobacterota* [85]. The universal absence of *Fibrobacterota* in all reactors by ammonia might have serious implications for cellulose decomposition during AD. Amongst these phyla, *Cloacimonadota* showed a markedly significant enhancement in the co-digesters compared with control-CM. The relative abundance of *Cloacimonadota* was slightly enhanced (3%) in the DFP mono-digestion, as compared to the inoculum, but it was significantly enhanced by about 8–14% in the treatment groups having 25, 50, and 75% DFP. The phylum *Cloacimonadota* is sensitive to high concentrations of ammonia nitrogen in the digester medium [86]. Organisms within the phylum *Cloacimonadota* are recognized as propionate oxidizers, and their stimulation in the co-digesters aligns with the minimal accumulation of propionate [87].

A comparison of the leading bacterial genera was conducted at the genus classification level to elucidate the impact of co-substrate DFP. At the genus level, the genera relative abundances enhanced include *Bacteroides* (9.4–42.1%), *Proteiniphilum* (4–12.2%), *W5* (4.1–11.8%), *Fermentimonas* (5.4–12.8%), and *Fonticella* (2.4–13.2%) (Figure 5b). *Bacteroidetes* can break down polysaccharides and oligosaccharides. The relative abundance of *Proteiniphilum* increased by approximately 12% in the DFP mono-digestion control and CM-50:DFP-50 co-digesters, whereas only a slight increase was observed in the CM-25:DFP-75 co-digesters; thus, these findings suggest that the genus *Proteiniphilum* likely functions to degrade protein [88]. Chen and Dong (2005) indicated that the genera *Caldicoprobacter* and *Proteiniphilum* were critical in the anaerobic co-digestion process of CM and corn straw [88]. *W5*, part of *Cloacimonetes*, was frequent, potentially aiding in the prevention of propionic acid accumulation [89]. *Fermentimonas* can enhance the breakdown of carbohydrates and complex proteins to generate acetic acid and H_2_ [90]. The higher abundance of *Fermentimonas* in co-digesters supports acetic acid formation, potentially contributing to the increased biogas yield (Figure 5b). It is apparent that *Fonticella* was also the dominant genus at co-digestion compared with the control-CM (Figure 5b). The abundance of *Fonticella* in treated reactors may explain the elevated acetic acid ratios, as this bacterium can metabolize bioavailable substrates (such as carbohydrates) in fermentation systems to produce acetic acid [91].

The analysis of bacterial phyla and genera demonstrates a clearly altered microbial structure that was formed in the co-digesters and mono-digesters. However, hydrolytic/acidogenic bacteria predominated in the treatment digesters compared to the control-CM.

#### 3.4.2. Archaeal Community

Anaerobic methanogenesis is the result of synergistic interactions among various bacterial and archaeal communities. The level of degradation of organic matter relies on the activity of these microorganisms, and the presence of a suitable co-substrate can optimize the microbial community [1]. A Venn diagram illustrates the patterns of archaeal OTU distributions (Figure 4d), showing that the OTUs that were shared by the mono- and co-digesters of CM and DFP were 31. The maximum and stable OTUs were observed in AcD reactor CM-75:DFP-25 (132), CM-50:DFP-50 (130), and CM-25:DFP-75 (117) compared with inoculum (108). The archaeal communities evolved, reflecting changes in AcD, which showed that adding the co-substrate DFP improved the diversity of the archaeal community in relation to the inoculum.

**Figure 6:**
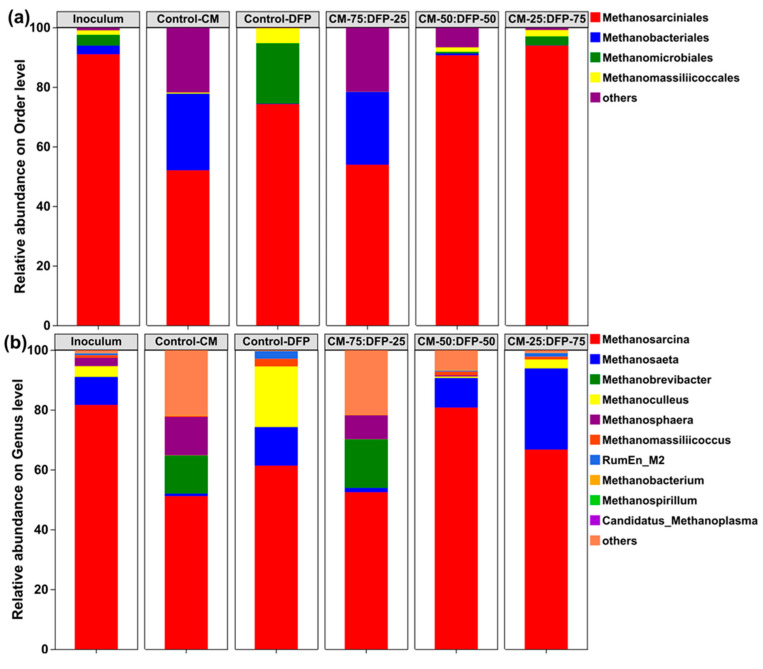
Archaeal community composition of the inoculum and mono- and co-digestion chicken manure (CM) and dragon fruit peel (DFP) at the archaeal order (**a**) and genus level (**b**).

The research explored the effect of various ratios of CM and DFP on the AD, focusing on the correlation between methane yield and microbial community composition. The diversity of archaeal flora was considerably altered by the presence of the DFP co-substrate. In archaeal communities categorized at the level of orders, *Methanosarciniales*, *Methanomicrobiales*, and *Methanobacteriales* remained the main methanogens, with a relatively high abundance across all treatment groups with CM and DFP of about 98% compared with control-CM (74%) and control-DFP (94%), indicating that acetoclastic and hydrogenotrophic methanogenesis were the pathways for methane conversion in the system (Figure 6a). Hydrogenotrophic methanogens, classified under the order *Methanomicrobiales*, are often found in environments with low pH, including acidogenic digestion systems and anaerobic digestate systems [90]. *Methanosarcinales*, conversely, are exclusively acetoclastic methanogens that directly generate methane from acetate [92]. Hydrogenotrophic methanogens (*Methanomicrobiales* and *Methanobacteriales)* utilize hydrogen derived from syntrophic acetate-oxidizing bacteria for methane production [93]. The abundances of order *Methanosarcinales* decreased by 40% in co-digestion CM-75:DFP-25 and mono-digestion CM, while their abundances increased in the co-digestion system CM-50:DFP-50 (130) and CM-25:DFP-75 (Figure 6a). The insignificant abundance of *Methanosarciniales* certainly attributed to the high concentration of NH_4_-N because the *Methanosarciniales* did not appear to be affected by higher levels of VFAs, owing to their capability to consume acetate, hydrogen, secondary alcohols, and methyl compounds as energy sources, consistent with the observation found in the literature [94]. It is important to note that a higher abundance of *Methanosarciniales* ensures a higher CH4 content in biogas. The AD process is driven by microbes, with the conversion of substrates into the desired metabolite product predominantly regulated by the metabolism of functioning anaerobic bacteria. For example, during the hydrolysis and acidogenic phases, fermentative microorganisms such as *Bacillota* and *Bacteroidetes* transformed intricate organic waste into VFAs, hydrogen, and CO_2_ [95]. Subsequently, the various categories of methanogens, including *Methanobacteriales*, *Methanosarcinales*, and *Methanomassiliicoccales*, may transform these generated organic acids and hydrogen into methane [96].

The methanogenic process comprises four principal pathways: carbon dioxide reduction, acetoclastic, methylotrophic, and methyl reduction pathways [97]. *Methanosarcina,* a facultative methanogen, may consume acetate, hydrogen, and carbon dioxide as substrates, displaying metabolic flexibility. The combined relative abundance of *Methanosarcina* and *Methanosaeta* was found to be highest in co-digesters CM-50:DFP-50 (90%) and CM-25:DFP-75 (94%), compared with mono-digestion controls (Figure 6b). *Methanosarcina* shows enhanced resilience to unfavorable environmental conditions and can utilize all four methanogenic pathways [98]. Strictly hydrogenotrophic genus *Methanoculleus*, about 22.3%, was significantly increased in the control-DFP and reduced in the CM control (Figure 6b).

Acetoclastic methanogenesis is known to be performed by two archaeal genera, *Methanosaeta* and *Methanosarcina*. Chen et al. (2017) observed that *Methanosaeta* populations are more competitive at low acetate levels (<1 mM) than *Methanosarcina* and vice versa at higher acetate concentrations [99]. Reports of inconsistent findings have indicated that *Methanosaeta* outnumber *Methanosarcina* at elevated acetate levels [99]. In this study, it is noteworthy that the methanogenic *Methanosaeta* became the dominant species in the CM-25:DFP-75 bioreactors, generating methane primarily from an acetate precursor [49]. The surprising competitiveness of *Methanosaeta* at elevated acetate levels was further supported by the enrichment of this genus under high acetate concentrations. This study demonstrated that the dominance of *Methanosaeta* populations in anaerobic digestion could be linked to their greater competitiveness at elevated acetate concentrations. Given the importance of acetoclastic methanogenesis in biological methane production, findings from this study could have major implications for developing strategies for more effective control of methanogenic treatment processes. The highest accumulative methane yield supports the idea that bacterial and archaeal organisms form a syntrophic relationship during acidogenesis and methanogenesis in the AcD system of CM DFP. *Methanosphaera* and *Methanobrevibacter* replaced the genus *Methanosarcina* in control-CM and CM-75:DFP-25. Through interspecies hydrogen transfer or interspecies formate transfer, the *Methanosphaera* species produce methane from H_2_ or formate [100]. The hydrogenotrophic methanogen pathway is the source of the enzymes associated with methane production in 55_bin.37, 53_bin.62, and 53_bin.4. This indicates that these species may possess the capability to generate hydrogenotrophic methane [101]. The *RumEn_M2* was improved in control-DFP and co-digesters CM-25:DFP-75 as compared with the control (Figure 6b). *RumEn_M2* has been identified as a novel H2-dependent methanogenic genus that exclusively utilizes methanol and methylamines for growth [102,103]. The addition of the co-substrate DFP prompted a clear shift in bacterial and archaeal community composition. This shift, likely facilitated by the improved nutrient balance, suggests an increase in microbial diversity and correlates with the observed enhancement in functional stability and methane production.

## 4. Conclusions

This study evaluated the influence of DFP as a co-substrate on optimizing the C/N ratio, improving the AD efficiency of CM, and maximizing bioenergy recovery. The findings confirm that co-digestion significantly enhances VFA conversion and maintains optimal pH levels, with the highest biomethane yield of 180.3 mL/g VS achieved at the CM-25:DFP-75 ratio, representing a 74.6% and 421.1% increase compared to mono-digestion of CM (103.3 mL/g VS) and DFP (34.6 mL/g VS), respectively. However, other treatment groups also delivered substantial improvements: CM-50:DFP-50 produced 148.3 mL/g VS (43.6% higher than CM), and CM-75:DFP-25 yielded 116.7 mL/g VS (12.9% higher than CM). Microbial community analysis revealed a marked enrichment of functional taxa critical for efficient AD, including hydrolytic bacteria (*Bacteroidetes*, *Bacillota*, and *Fibrobacterota*) and acetoclastic methanogens (*Methanosarcina* and *Methanosaeta*), which collectively drive substrate degradation and methane production. While biomethane production increased with higher DFP ratios, the scalability of the proposed co-digestion strategy faces a key limitation of supply chain constraint. The seasonal availability (tied to tropical fruit harvesting cycles) and geographic concentration of the production of DF may limit consistent feedstock access for large-scale biogas facilities. Furthermore, to validate real-world applicability, pilot-scale trials are needed to focus on testing DFP-CM co-digestion under semi-continuous operational conditions to assess long-term stability and methane yield consistency, evaluating feedstock preprocessing strategies, and combining the use of DFP-CM with other carbon/nitrogen-rich residues without any supply chain constraints for effective bioenergy recovery. Consequently, this study was a pioneer effort to provide baseline data for the potential of DFP for use as a co-substrate to reduce fossil fuel dependency, effectively manage waste, and contribute to circular economy goals in the agriculture sector.

## Figures and Tables

**Table 1:** The physicochemical characteristics of the anaerobically digested sludge (ADS), chicken manure (CM), and dragon fruit peel (DFP) that were used in the present investigation.

Analysis	ADS	CM	DFP
Proximate analysis (wt.%)			
TS	6.4 ± 0.9	56.1 ± 1.4	35.8 ± 1.1
Moisture	93.6 ± 1.1	68.2 ± 1.3	64.2 ± 2.4
VS	4.1 ± 0.4	44.9 ± 1.1	23.2 ± 1.0
Ash	1.2 ± 0.1	22.9 ± 0.2	8.9 ± 0.2
Fixed carbon	10.6 ± 0.5	8.9 ± 1.0	4.1 ± 0.3
Ultimate analysis (wt.%)			
Carbon	31.9 ± 1.3	39.8 ± 0.3	44.3 ± 1.3
Nitrogen	1.3 ± 0.5	3.0 ± 0.2	1.1 ± 0.4
Hydrogen	4.5 ± 1.34	4.1 ± 0.5	9.1 ± 0.5
Sulphur	1.5 ± 0.7	0.5 ± 0.3	0.9 ± 0.1
Oxygen	60.8 ± 1.5	54.0 ± 1.2	49.8 ± 1.6
C/N ratio	24.5	13.2	40.3
Physical properties			
pH	7.2	7.4	n.d

**Table 2:** Total biogas and methane produced and kinetic parameters of mono-digestion (CM and DFP) and co-digestion of CM and DFP by the Modified Gompertz Model.

	Mono- and Co-Digestion Reactors’ Efficiency of CM and DFP		Kinetics (Modified Gompertz Model)		
Reactor ID	Biogas/Biomethane Production (mL/g VS)	M_max_ (mL/g VS)	R_m_ (mL/g VS)	*λ* (Days)	R^2^
Biogas					
Control-CM	276.8 ± 2	275	~0.008e	0.347	0.997
Control-DFP	190.1 ± 1	194	~0.470e	0.287	0.998
CM-75:DFP-25	325.5 ± 2	337	~2.459e	0.316	0.992
CM-50:DFP-50	366.3 ± 2	392	~0.008e	0.242	0.994
CM-25:DFP-75	411.2 ± 3	433	~0.864e	0.196	0.998
Biomethane					
Control-CM	103.3 ± 1	106	~7.545e	0.559	0.995
Control-DFP	34.6 ± 1	36	~7.490e	0.366	0.988
CM-75:DFP-25	116.7 ± 2	118	~2.464e	0.469	0.997
CM-50:DFP-50	148.2 ± 1	150	~1.706e	0.369	0.996
CM-25:DFP-75	180.3 ± 2	181	~0.001e	0.241	0.997

## Data Availability

The data used in this research article have been presented here and are supported by the Supplementary Materials Section.

## Supplementary Materials

The following supporting information can be downloaded at https://www.mdpi.com/article/10.3390/biology15010083/s1, Figure S1: Variation in pH of CM and DFP mono- and co-digestion (a and b); Table S1: Batch anaerobic digestion assays. Each assay was run in triplicate.

## Abbreviations

AD: anaerobic digestion, AcD: anaerobic co-digestion, DF: dragon fruit, DFP: dragon fruit peel, CM: chicken manure, VFAs: volatile fatty acids, C/N: carbon–nitrogen ratio, TS: total solid, VS: volatile solid, OTUs: operational taxonomic units.
