# Supplementary material for: Enhanced Biomethane Conversion and Microbial Community Shift Using Anaerobic/Mesophilic Co-Digestion of Dragon Fruit Peel and Chicken Manure
Source: Biology (Basel). 2025 Dec 31;15(1):83. doi: 10.3390/biology15010083 (PMC12785068; doi:10.3390/biology15010083)
Supplement: Supplementary file 1 [file biology-15-00083-s001.zip › biology-4022390-supplementary.pdf]

SUPPLEMENTARY MATERIALS

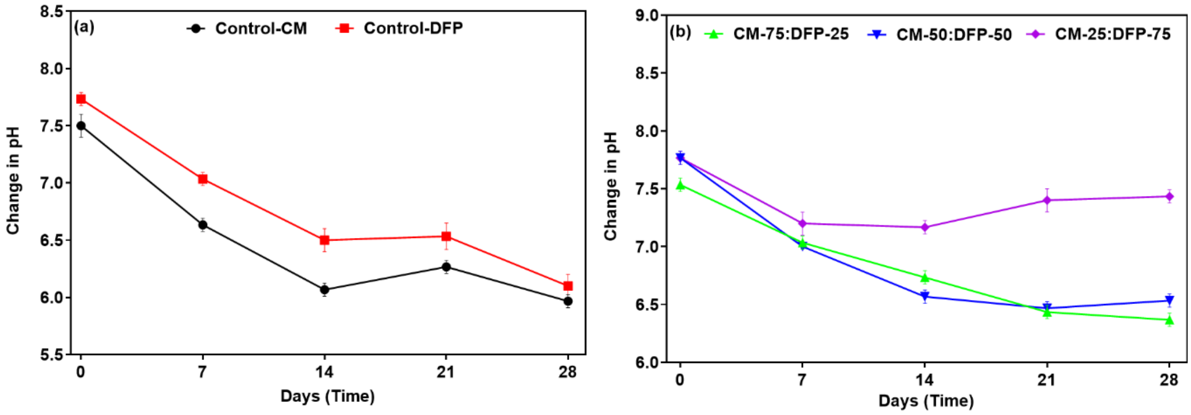

Figure S1. Variation in pH of CM and DFP mono- and co-digestion (a and b).

Table S1. Batch anaerobic digestion assays. Each assay was run in triplicate.

Setup	ADS (mL)	CM g/L	DFP g/L	Reactor WV	Headspace mL	C:N	Reactor VS g/L	S/I Ratio (VS Basis)
**Mono-digestion (Control)**								
**Control-CM**	120	15	0	350	150	13.2	4.6	1.4
**Control-DFP**	120	0	15	350	150	40.3	2.7	0.7
**Co-digestion (Treatments)**								
**CM-75:DFP-25**	120	11.7	3.3	350	150	15.1	4.1	1.2
**CM-50:DFP-50**	120	7.5	7.5	350	150	18.5	3.6	1.0
**CM-25:DFP-75**	120	3.3	11.7	350	150	24.9	3.1	0.8

W/V: Working volume
